# Mitochondrial dysfunction and impaired growth of glioblastoma cell lines caused by antimicrobial agents inducing ferroptosis under glucose starvation

**DOI:** 10.1038/s41389-022-00437-z

**Published:** 2022-10-04

**Authors:** Kenji Miki, Mikako Yagi, Koji Yoshimoto, Dongchon Kang, Takeshi Uchiumi

**Affiliations:** 1 Department of Clinical Chemistry and Laboratory Medicine, Graduate School of Medical Sciences, Kyushu University, Higashi-ku, Fukuoka, 812-8582 Japan; 2 Department of Neurosurgery, Graduate School of Medical Sciences, Kyushu University, Higashi-ku, Fukuoka, 812-8582 Japan; 3 Department of Health Sciences, Graduate School of Medical Sciences, Kyushu University, Higashi-ku, Fukuoka, 812-8582 Japan

**Keywords:** CNS cancer, Apoptosis

## Abstract

Glioblastoma is a difficult-to-cure disease owing to its malignancy. Under normal circumstances, cancer is dependent on the glycolytic system for growth, and mitochondrial oxidative phosphorylation (OXPHOS) is not well utilized. Here, we investigated the efficacy of mitochondria-targeted glioblastoma therapy in cell lines including U87MG, LN229, U373, T98G, and two patient-derived stem-like cells. When glioblastoma cells were exposed to a glucose-starved condition (100 mg/l), they rely on mitochondrial OXPHOS for growth, and mitochondrial translation product production is enhanced. Under these circumstances, drugs that inhibit mitochondrial translation, called antimicrobial agents, can cause mitochondrial dysfunction and thus can serve as a therapeutic option for glioblastoma. Antimicrobial agents activated the nuclear factor erythroid 2-related factor 2–Kelch-like ECH-associated protein 1 pathway, resulting in increased expression of heme oxygenase-1. Accumulation of lipid peroxides resulted from the accumulation of divalent iron, and cell death occurred via ferroptosis. In conclusion, mitochondrial OXPHOS is upregulated in glioblastoma upon glucose starvation. Under this condition, antimicrobial agents cause cell death via ferroptosis. The findings hold promise for the treatment of glioblastoma.

## Introduction

Glioblastoma is the most dreadful disease among malignant brain tumors, with an extremely poor prognosis [1–3]. Glioblastoma cannot be completely cured, causing the death of patients within a few years of diagnosis [3, 4]. Several studies on its treatment courses have been conducted; only temozolomide (TMZ) is promising as a standard treatment option, despite prolonging the overall survival by only 2.5 months [3]. However, glioblastoma cells usually become resistant to TMZ [5, 6].

Mitochondria are responsible for the production of adenosine triphosphate by oxidative phosphorylation (OXPHOS) and play an important role in Ca buffering, β-oxidation, reactive oxygen species (ROS) production, and apoptosis [7]. Additionally, mitochondria are associated with various diseases, including cancer [8]. Tumor cells are dependent on aerobic glycolysis for producing cellular energy [9]. However, recent studies have shown that increased OXPHOS activity in cancer cells [10], especially in cancer stem cells (CSCs), results in an elevated mitochondrial oxygen consumption rate (OCR) [11]. CSCs play a critical role in various types of cancers [12, 13], and malignant tumors are heterogenous and contain CSCs [14]. Research on the relationship between mitochondria and glioblastoma has shown that mitochondria play an important role in inducing TMZ resistance [15].

Previously, we reported the importance of mitochondria in cancer [16, 17]; particularly, we found that oncogenic HRAS indirectly suppresses the mitochondrial OCR, but oxygen consumption is essential for tumorigenesis [17]. Targeting mitochondria is considered to be effective in cancer treatment because of the dependence of cancerous tissues on mitochondria [17, 18].

Lamb et al. reported that antibiotics that target mitochondria are effective against various cancer cells. Twelve different cancer cell lines were used, and antibiotic drugs were successfully used to eliminate CSCs. Many antibiotic drugs are non-toxic to normal cells and are likely to reduce the adverse effects of anti-cancer therapy [18]. Matsumoto et al. reported that doxycycline (DOXY) is effective in targeting mitochondria in CSC-like cells in prostate cancer [16]. Chloramphenicol (CAP) and DOXY are some of the main drugs that cause mitochondrial dysfunction. CAP binds directly to the A-site crevice on the 50S ribosomal subunit and causes translation inhibition [19].

To make mitochondrial OXPHOS-dominant condition, cells have been subjected to serum starvation, leading to upregulated mitochondrial OXPHOS [17]. Serum starvation is a common feature of solid tumors during anti-angiogenesis, irradiation, and chemotherapy [17, 20]. Under low-nutrient conditions, antibiotics are effective because of the dependence of cells on the mitochondria. However, to our knowledge, there are no reports on the treatment of mitochondrial dysfunction under glucose-starved conditions.

Although Matsumoto et al. reported that DOXY causes cell death via apoptosis, there are numerous pathways, besides apoptosis, that lead to cell death, and little is known about the mechanism of cell death caused by antibiotics [16]. One of these pathways that has been attracting attention is ferroptosis [21]. Ferroptosis has several regulatory pathways, including the transferrin receptor (TFRC)–six transmembrane epithelial antigens of the prostate 3 (STEAP3)-divalent metal transporter 1 (DMT1) pathway, xCT-glutathione peroxidase 4 (GPX4) pathway, and Kelch-like ECH-associated protein 1 (KEAP1)–nuclear factor erythroid 2-related factor 2 (NRF2)–heme oxygenase-1 (HO-1) pathway [21].

KEAP1 is a repressor of NRF2 activity and plays a key role in its regulation [21, 22]. HO-1 level is elevated under NRF2 upregulation [21], and its activation increases labile Fe^2+^, leading to ROS overload [23]. Upstream of this pathway, p62, which is related to autophagy and mitochondria, regulates the expression of KEAP1 [24]. Although little is known about ferroptosis, we suspect that this pathway may be related to mitochondrial function.

Abnormal iron distribution and content disrupts normal physiological processes [21]. The accumulation of iron-dependent lipid ROS is involved in ferroptosis [21]. Polyunsaturated fatty acids are sensitive to lipid peroxidation and are essential for ferroptosis [25]. Different methods to prove the occurrence of ferroptosis have been reported [26]. However, we believe that it is difficult to determine ferroptosis occurrence using just western blotting or mRNA expression, necessitating more specific methods for its detection.

Here, we investigated the efficacy of mitochondrial dysfunction under conditions of glucose starvation for glioblastoma therapy. We examined the dominance of OXPHOS under conditions of glucose starvation and whether CAP, which causes mitochondrial dysfunction, is effective. Additionally, we investigated how CAP suppresses cell proliferation via ferroptosis, as there are no reports on treatments that induce mitochondrial dysfunction under glucose starvation and the mechanism of cell death caused by CAP.

## Results

### Increased expression of COX I and II in U87 and LN229 via glucose starvation

**Fig. 1:**
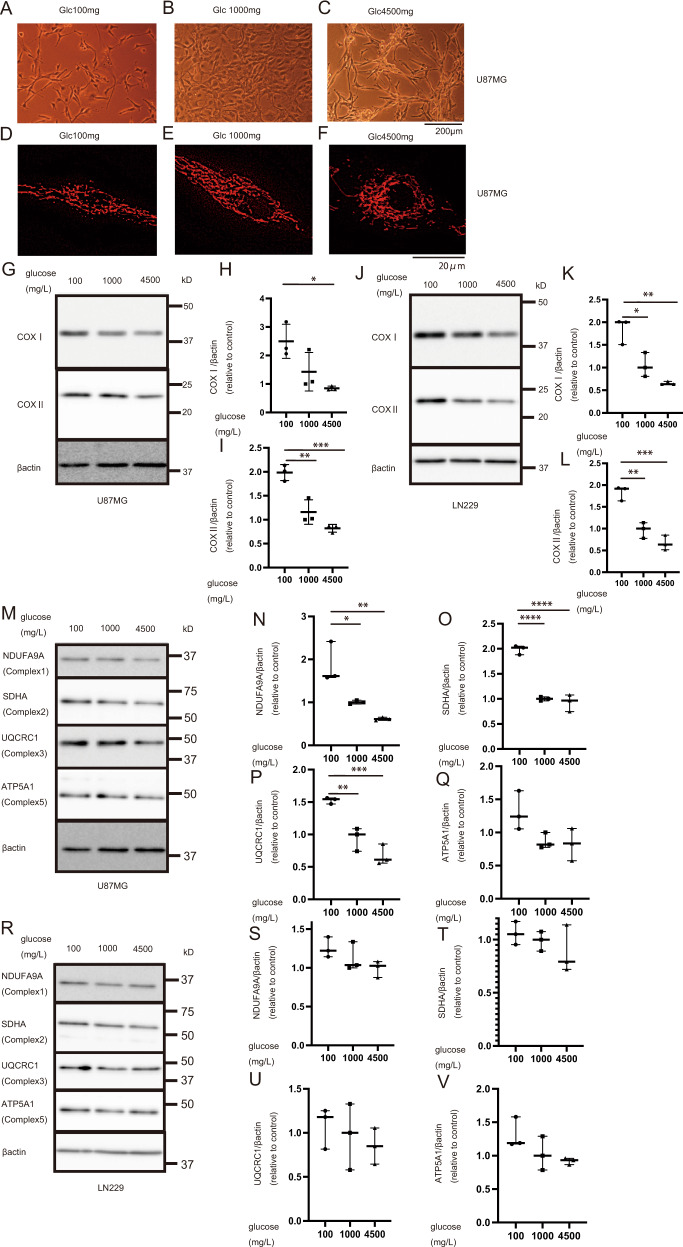
Effects of glucose on morphology and OXPHOS proteins. U87 cells under different glucose concentrations (**A** 100 mg/l; **B** 1000 mg/l; **C** 4500 mg/l). U87 cells were cultured for 3 days under different glucose concentrations and stained with MitoTracker Red (**D** 100 mg/l; **E** 1000 mg/l; **F** 4500 mg/l). **G** Western blot of mtDNA-encoded COX I and COX II in U87 under glucose starvation after 7-day culture. β-Actin was used as an internal control. **H**, **I** Quantification of COX I and COX II in U87. **J** Western blot of COX I and COX II in LN229 under glucose starvation after 7-day culture and (**K**, **L**) quantification results. **M** Western blot of nuclear-encoded OXPHOS proteins, NDUFA9A, SDHA, UQCRC1, and ATP5A in U87 and (**N**–**Q**) quantification results (*N* = 3). Glucose starvation tends to cause high expression of these proteins after 7-day culture. **R** Western blot of LN229 and (**S**–**V**) quantification results. Values are presented as mean ± SD (*N* = 3). Ordinary one-way ANOVA with Tukey’s multiple comparisons test was performed for glucose 100 mg/l vs. 1000 mg/l and glucose 100 mg/l vs. 4500 mg/l. *p < 0.05, **p < 0.01, ***p < 0.001, ****p < 0.0001.

Mitochondrial OXPHOS becomes dominant under serum starvation or low-nutrient conditions in cancer cells [17]. To simplify the conditions, we focused on glucose because cancer cells may be more dependent on OXPHOS under lower-glucose conditions to efficiently produce more ATP. U87MG(U87) and LN229 sub-cultured with a high glucose concentration (4500 mg/l) were seeded in culture media with reduced glucose concentrations to investigate the effects of glucose concentration on mitochondria in the glioblastoma cells. The general cell morphologies of U87 were altered under lower-glucose concentrations without apparent structural changes in mitochondria (Fig. 1A–F). Under lower glucose concentration, the cells were wide in shape and proliferated without stacking. Conversely, the cells were piled up during proliferation under high glucose concentration (Fig. 1B, C). To investigate mitochondrial morphology, Huang et al. [27] used mito-GFP, and Rambold et al. [28] classified mitochondria into tubules and fragments. Here, under difference glucose concentrations, the ratio of tubular mitochondria did not increase and the expression of proteins related to mitochondrial morphology including OPA1 and MFN2 did not change (Fig. 1D–F and Supplementary Fig. 1A–C).

It has been reported that OXPHOS becomes dominant under low-nutrient conditions [17]. Therefore, we analyzed the OXPHOS proteins. Glucose starvation is expected to induce efficient glucose utilization, that is, OXPHOS. As expected, the expression of COX I and COX II, which are encoded by mitochondrial DNA, was elevated under glucose starvation for 7 days (Fig. 1G–L), and that of NDUFA9A (Complex1), SDHA (Complex2), and UQCRC1 (Complex3) encoded by nuclear genes was also elevated, especially in U87 cells (Fig. 1M–V). We suggest that glucose at a low concentration activates OXPHOS and upregulates OXPHOS proteins. In addition, the *PGC1α* mRNA level was increased under glucose-starved condition and the *COX I* and *II* mRNA levels were decrease under glucose-starved condition (Supplementary Fig. 1D–F). These results imply that the increased expression of COX proteins is related to mitochondrial translation.

### Increased activity of OXPHOS in U87 and LN229 under hypoglycemic condition

**Fig. 2:**
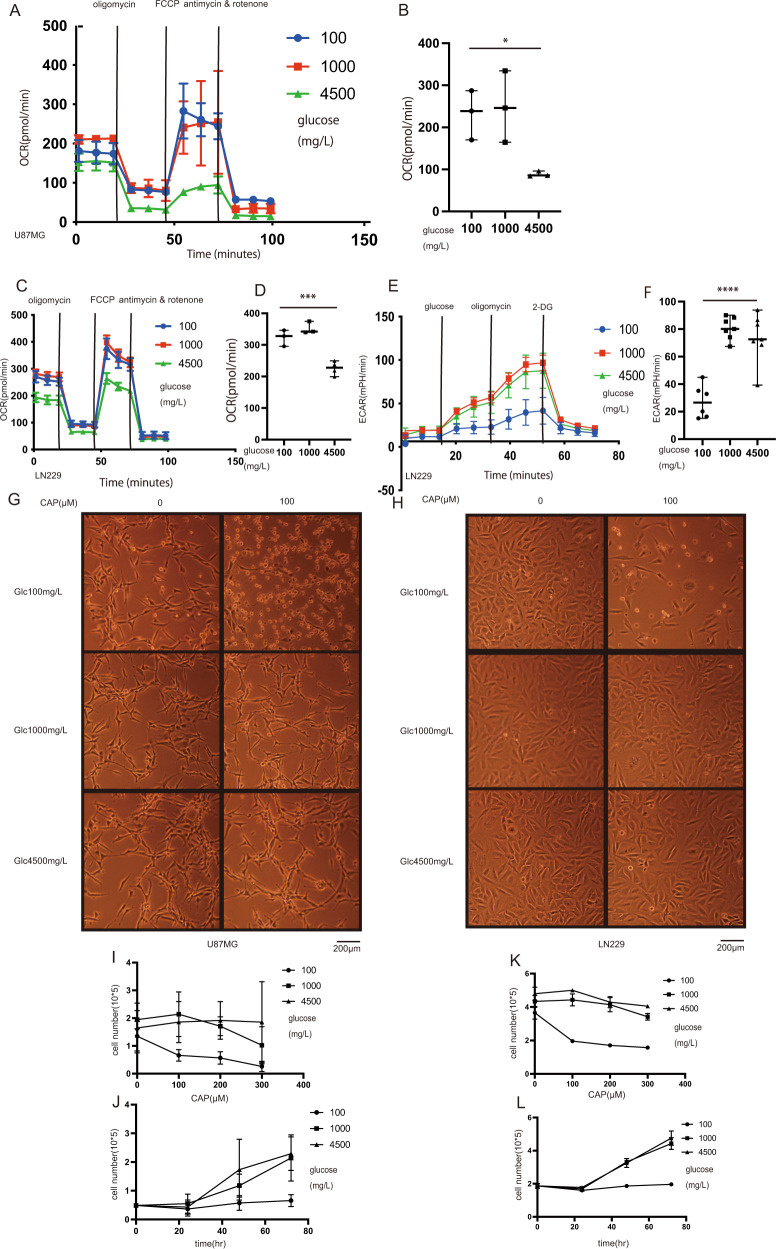
Effects of glucose concentration on OXPHOS and effects of antimicrobial agents under glucose-starved condition. **A** Traces of OCR in U87 under different glucose concentrations and **B** quantification after 3-day culture (*N* = 3). **C** Traces of OCR in LN229 and **D** quantification after 3-day culture (*N* = 3 or 4). **E** Traces of ECAR in LN229 after 7-day culture and **F** quantification results (*N* = 6 or 7). Values are presented as mean ± SD. Statistical significance was assessed using the ordinary one-way ANOVA test with Tukey’s multiple comparisons test performed on glucose 100 mg/l vs. 1000 mg/l and glucose 100 mg/l vs. 4500 mg/l. *p < 0.05, ***p < 0.001, ****p < 0.0001. **G** The relationship between CAP and glucose concentration in U87. CAP was effective at 100 mg/l glucose. **H** The relationship between CAP and glucose concentration in LN229. CAP was effective at glucose 100 mg/l. **I** Growth curve of CAP dose-dependency and each glucose condition in U87 (cells were seeded in a 12-well dish and counted using trypan blue) (*N* = 3 or 4). **J** Time-course of growth curve with CAP 100 μM with each glucose concentration in U87 (*N* = 3 or 4). **K** Survival curve of CAP dose-dependency and each glucose condition in LN229 (cells were seeded in a 6-well dish and counted using a Coulter counter) (*N* = 3). **L** Time-course of survival curve with CAP 100 μM with each glucose concentration in LN229 (*N* = 3).

We monitored the OCR to evaluate OXPHOS [29]. The OCR was elevated at 100 and 1000 mg/l glucose concentrations compared to that at 4500 mg/l (Fig. 2A–D). After the addition of oligomycin, ECAR showed that LN229 cells had more glycolysis reserve at 1000 and 4500 mg/l glucose concentrations (Fig. 2E, F). Conversely, at 100 mg/l glucose concentration, cells no longer relied on glycolysis but on the tricarboxylic acid cycle. Considering these data, glioblastoma cells relied mainly on mitochondria at a glucose concentration of 100 mg/l. Conversely, under hyperglycemic conditions, cells relied primarily on glycolysis. At 1000 mg/l glucose concentration, glioblastoma cells could use both OXPHOS and glycolysis. Our findings demonstrated that hypoglycemic conditions made glioblastoma cells dependent on mitochondria, suggesting that the inhibition of OXPHOS with glucose starvation can be a form of treatment.

### CAP can be a treatment for glioblastoma

The above results clearly raised the possibility that mitochondria could be a good target for glioblastoma treatment under certain situations. CAP and DOXY are antibiotics that cause mitochondrial dysfunction with inhibition of ribosome activity [30–32]. In U87 cells, CAP at concentrations of 10 and 100 μM induced cell death at 100 mg/l glucose (Fig. 2G). Similarly, 100 μM CAP caused cell death in the LN229 cell line (Fig. 2H). The growth curves of CAP dose-dependency and time-course (Fig. 2I–L) showed that cell death was caused only at a glucose concentration of 100 mg/l but not at concentrations of 1000 or 4500 mg/l (see also Supplementary Fig. S2A–D). In U373 and T98G, the same results were obtained (Supplementary Fig. S2E, F). These findings show that CAP was effective only at a glucose concentration of 100 mg/l where cells are mostly dependent on OXPHOS. DOXY was also effective at a glucose concentration of 100 mg/l (Supplementary Fig. S2G, H).

### CAP causes cell death, not via the apoptosis pathway

The observation of cell death induced with CAP or DOXY under glucose starvation prompted us to elucidate the underlying molecular mechanisms. There are no reports on treatment using medicines, including CAP and DOXY, that induce mitochondrial dysfunction under glucose starvation. Matsumoto et al. reported that DOXY caused apoptosis in prostate cancer via endoplasmic reticulum stress, partially causing ATF4 induction [16]. First, we speculated the same pathways and then checked apoptosis pathways. However, CAP did not upregulate ATF4 expression, decreased caspase-3 level, or elevated p53 upregulated modulator of apoptosis. No cleaved caspase-3 expression was detected in glioblastoma cells (Supplementary Fig. 3A–D). We conclude that CAP did not cause apoptosis under the conditions described herein; therefore, CAP may cause cell death via other pathways. Moreover, no change in mixed lineage kinase domain-like protein mRNA level was observed, which implies that the induction of the necroptosis pathway and Necrstatin-1, an inhibitor of necroptosis, did not prevent cell death. In addition, no changes were observed in the levels of ferredoxin 1 (FDX1), which is a key protein of the cuproptosis pathway and ammonium tetratiomolybdate, an inhibitor of cuproptosis, and they did not rescue the cell death (Supplementary Fig. 3E–I). In LN229, the same results were obtained for the apoptosis pathway (Supplementary Fig. S4A–D).

### CAP causes cell death via the ferroptosis pathway

**Fig. 3:**
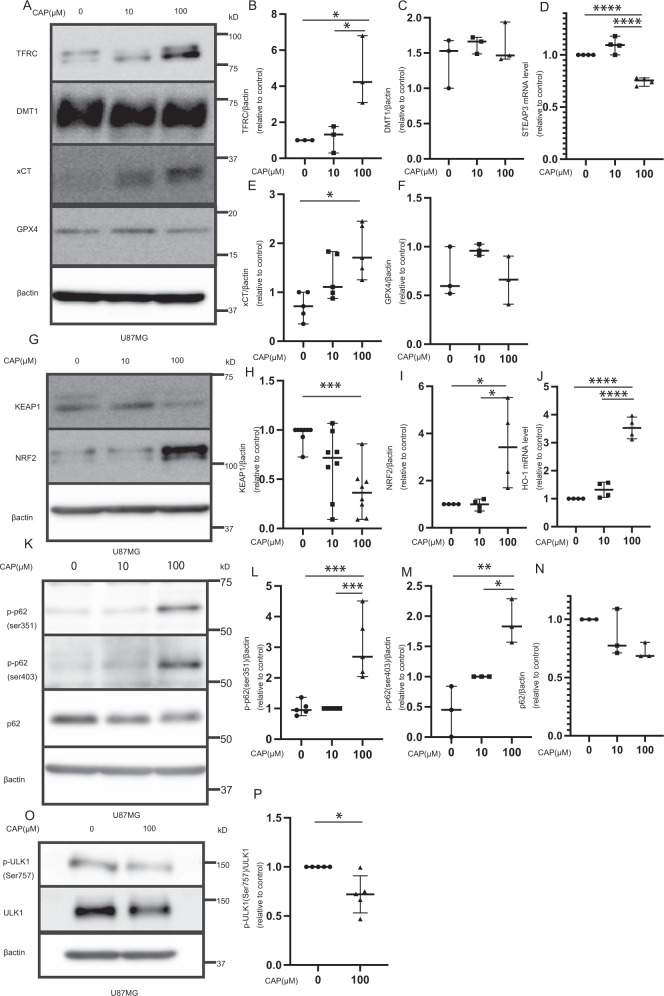
KEAP 1-NRF 2-HO-1 pathways are activated. A change in ferroptosis was observed after CAP treatment for 3 days in U87. **A** Western blot revealed that the TFRC level (*N* = 3) was changed, but the DMT1 (*N* = 3), xCT (*N* = 5), and GPX4 (*N* = 3) levels were not changed after CAP treatment and (**B**, **C**, **E**, **F**) quantification results. **D** Quantification of STEAP3 mRNA expression (*N* = 4). **G** Western blot revealed that the KEAP1 (*N* = 7) and NRF2 (*N* = 4) levels were changed and **H**, **I** quantification results. **J** Quantification of HO-1 mRNA expression (*N* = 4). **K** After CAP treatment, western blotting of p-p62 ser 351, 403, and p62 was performed and **L**–**N** p-p62 ser 351 (*N* = 5), 403 (*N* = 3), and p62 (*N* = 3) were quantified. **O** Western blot revealed that the p-ULK1 (Ser757) were changed and **P** quantification results (*N* = 5). Values are presented as mean ± SD. Student’s *t* test (**P**) or ordinary one-way ANOVA with Tukey’s multiple comparisons test (**B**–**F**, **H**–**J**, **L**–**N**) was performed on control vs. CAP 10 μM and control vs. CAP 100 μM. *p < 0.05, ***p < 0.001, ****p < 0.0001.

Recently, ferroptosis was discovered as a new cell death mode [21]. We evaluated whether ferroptosis occurred during our experiments. Ferroptosis has several underlying pathways, including the TFRC–STEAP3–DMT1 pathway, xCT–GPX4 pathway, and KEAP1–NRF2–HO-1 pathway [21]. Each pathway was examined to determine whether ferroptosis was induced by CAP. First, in the case of the TFRC–STEAP3–DMT1 pathway, TFRC expression was found to be elevated after CAP use; however, the STEAP3 and DMT1 levels were not elevated (Fig. 3A–D and Supplementary Fig. S5A–D). Second, xCT was elevated only in U87; however, no change was detected in LN229, and GPX4 was unchanged by CAP (Fig. 3A, E, F and Supplementary Fig. S5A, E–G). Finally, the KEAP1–NRF2–HO1 pathway was elucidated. CAP downregulated KEAP1 in both cell lines. KEAP1 has been reported to inhibit NRF2 expression [21, 33, 34]; CAP elevated NRF2, which enhanced HO-1 expression. Expectedly, CAP also elevated HO-1 expression at the mRNA level (Fig. 3G–J and Supplementary Fig. S5H–K). Therefore, these results suggest that CAP causes cell death via the ferroptosis pathways, especially the KEAP1–NRF2–HO-1 pathway.

Next, we elucidated how KEAP1 expression is decreased. Reportedly, p62 regulates KEAP1 expression; especially, phospho p62 (p-p62) regulates KEAP1 expression [35]. Therefore, we suspected that p-p62 might have a major role in this pathway. Phospho-p62 ser 351 and ser 403 were analyzed. In both cell lines, CAP elevated both p-p62 ser 351 and ser 403 (Fig. 3K–M and Supplementary Fig. S5L–N). Ichimura et al. reported that p62 was repressed by autophagy [35]. CAP downregulated p62 expression slightly in the U87 cell line (Fig. 3N) but the expression did not change in the LN229 cell line (Supplementary Fig. S5O). These results suggest that CAP first elevates the expression of p-p62; subsequently, KEAP1 is lowered and NRF2 is elevated, and then finally, HO-1 is elevated. Upstream of p-p62, there was no apparent change in AMP-activated protein kinase (AMPK) levels by CAP (Supplementary Fig. 6A–C). However, levels of p-unc-51 like autophagy activating kinase 1 (ULK1) (Ser757) which inactivates ULK1 decreased (Fig. 3O, P). Regarding the effect of CAP on mitochondrial morphology and mitophagy, the OPA1 and MFN2 levels did not change, suggesting no apparent change in mitochondrial morphology. Regarding mitophagy, although the PINK1 level did not change, the LC-3 level decreased after CAP injection; this finding may imply the possibility of decrease in autophagy (Supplementary Fig. 6D–H). This is consistent with those from previous studies that state mitochondrial damage causes changes in autophagy [36]. Next, we investigated further events after HO-1 elevation and whether they led to ferroptosis.

### CAP induces the production of lipid radicals via the ferroptosis pathway

**Fig. 4:**
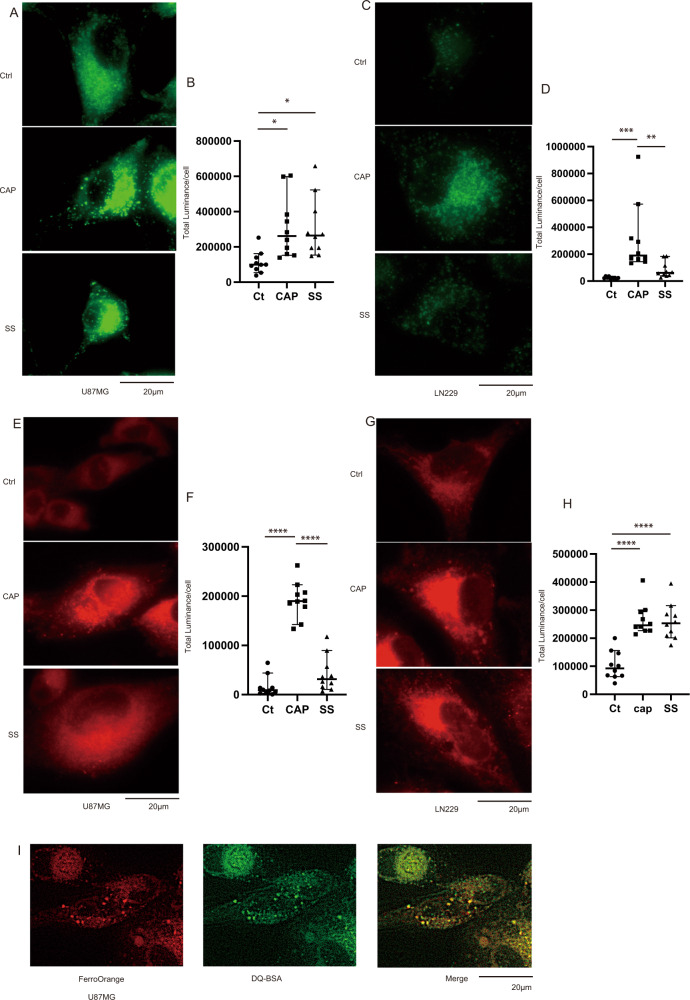
Induction of lipid radical accumulation by CAP. **A** In U87, LipiRADICAL luminance increased after treatment with CAP and SS (observed after 72 h) and **B** quantification of total luminance of LipiRADICAL Green. **C** LipiRADICAL luminance showed an increase in the CAP and SS groups in LN229 and **D** quantification of total luminance of LipiRADICAL Green. **E** Luminance of FerroOrange showed an increase in the CAP and SS groups in U87 and **F** quantification of total luminance of FerroOrange. **G** Luminance of FerroOrange showed an increase in the CAP and SS groups in LN229 and **H** quantification of total luminance of FerroOrange. **I** In U87, the luminance of FerroOrange and DQ-BSA colocalized. Values are presented as mean ± SD (*N* = 10). Ordinary one-way ANOVA with Tukey’s multiple comparisons test was performed on control vs. CAP and control vs. SS. *p < 0.05, **p < 0.01, ***p < 0.001, ****p < 0.0001.

As elevation in the above-mentioned proteins was related to iron homeostasis, we evaluated the effect of CAP under glucose starvation on the induction of ferroptosis. We searched for an effective method to detect ferroptosis. Ferroptosis is caused by iron-mediated lipid peroxidation [37]. Accordingly, we investigated a method to detect lipid peroxidation and intracellular iron levels. To establish that CAP causes ferroptosis, LipiRADICAL Green and FerroOrange were used. LipiRADICAL Green detects lipid radicals, a ferroptosis marker. FerroOrange detects intracellular free ferrous ion (Fe^2+^). We used sodium selenite (SS), reported to cause ferroptosis, as a positive control [38]. In both cell lines, CAP and SS elevated the LipiRADICAL Green’s total luminance (Fig. 4A–D). CAP and SS elevated FerroOrange signals or Fe^2+^ level (Fig. 4E–H). Interestingly, the FerroOrange luminance coincided with that of lysosome detected with DQ-BSA, suggesting lysosomal Fe accumulation (Fig. 4I). These results suggest that CAP increases intracellular Fe^2+^ level and in turn elevates lipid radicals, a marker of ferroptosis.

### DFO and ZnPPIX inhibit ferroptosis caused by CAP

**Fig. 5:**
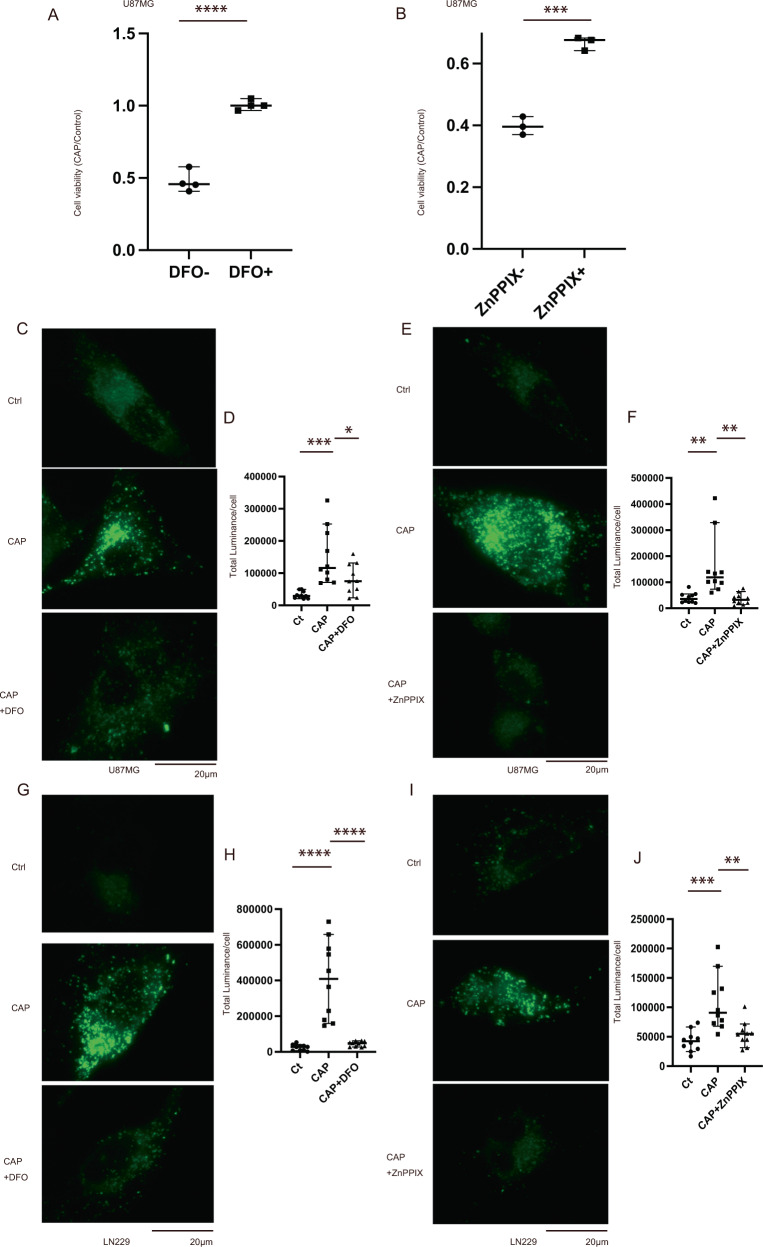
Inhibition of ferroptosis by DFO and ZnPPIX. **A**, **B** In U87, cell viability assay revealed that CAP and DFO or ZnPPIX reduced cell death after 3 days. Initially, 50 μM DFO was injected, and then, 25 μM DFO was added at 48 h or initially, 2.5 μM ZnPPIX was injected. Values are presented as median (*N* = 3). Student’s *t* test was performed. ***p < 0.001, ****p < 0.0001. **C** LipiRADICAL showed that DFO inhibited the effect of CAP in U87 (observed after 72 h) and **D** quantification of total luminance of LipiRADICAL Green. **E** LipiRADICAL showed that ZnPPIX inhibited the effect of CAP in U87 (observed after 48 h) and **F** quantification of total luminance of LipiRADICAL Green. **G** LipiRADICAL showed the DFO-inhibited CAP’s effect in LN229 (observed after 24 h) and **H** quantification of total luminance of LipiRADICAL Green. **I** LipiRADICAL showed that ZnPPIX inhibited the effect of CAP (observed after 48 h) and **J** quantification of total luminance of LipiRADICAL Green. Values are presented as mean ± SD (*N* = 10). Ordinary one-way ANOVA with Tukey’s multiple comparisons test was performed. *p < 0.05, **p < 0.01, ***p < 0.001, ****p < 0.0001.

Next, we examined whether the elevated Fe^2+^ and lipid radicals induce ferroptosis. Here, we considered that iron accumulation and HO-1 elevation may be the key factors in the ferroptosis pathway induced by CAP. Two inhibitors were used: DFO as an iron chelator and ZnPPIX as an HO-1 inhibitor. DFO and ZnPPIX also inhibited the CAP-induced cell death (Fig. 5A, B and Supplementary Fig. S7A, B). And DFO and ZnPPIX suppressed the CAP-induced lipid radical accumulation (Fig. 5C–J). Considering DFO inhibited CAP-induced cell death completely but ZnPPIX inhibited cell death sufficiently but not completely. Taken together, under CAP treatment, the KEAP1–NRF2 pathway is one of the major pathways that lead to the accumulation of intracellular Fe^2+^ and lipid radicals. Furthermore, glucose starvation made glioblastoma cells mitochondrial OXPHOS dependent, through which CAP could effectively inhibit cell growth via the ferroptosis pathway.

### CAP can cause cell death in patient-derived stem-like cells

**Fig. 6:**
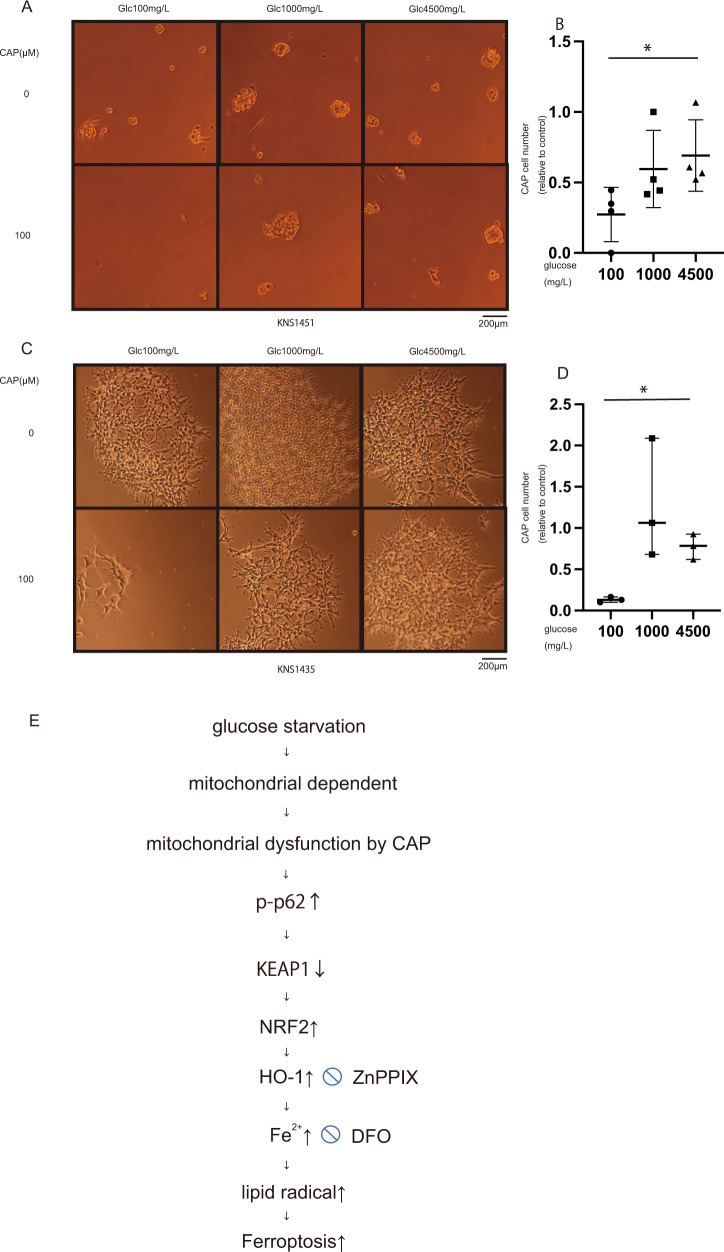
Effects of CAP in patient-derived stem-like cells. **A** Relationship between CAP and glucose concentration in KNS1451. CAP was effective at a glucose concentration of 100 mg/l. **B** Cell viability with CAP under glucose concentrations of 100, 1000, and 4500 mg/l relative to the control showed that CAP was effective under glucose-starved conditions. Values are presented as mean ± SD. Student’s *t* test was performed on glucose 100, 1000, and 4500 mg/l (*N* = 4), *p < 0.05. **C** Relationship between CAP and glucose concentration in KNS1435. CAP was effective under a glucose concentration of 100 mg/l. **D** Cell viability with CAP under glucose concentrations of 100 mg/l and 4500 mg/l relative to the control showed that CAP was effective under glucose-starved conditions after 9 days. Values are presented as mean ± SD. Ordinary one-way ANOVA test was performed (*N* = 3). *p < 0.05. **E** This scheme shows our proposed novel mechanism for this study.

Our results so far were obtained through experimentation on only commonly available cell lines. To emphasize the importance of the findings, we performed an in vitro study using patient-derived stem-like cells (KNS1451 and 1435) established by our group, because these stem-like cells derived from patients can form a sphere that mimics a tumor. The upper panel in Fig. 6A (0 μM CAP) shows the sphere formation. CAP induced the death of the stem-like cells under glucose starvation. However, under hyperglycemia, CAP had little influence (Fig. 6A–D). These were consistent with the results obtained in U87, LN229, U373, and T98G. Therefore, we suggest that inducing mitochondrial OXPHOS impairment via glucose starvation can be an effective treatment for glioblastoma, even in vivo.

## Discussion

We attempted glioblastoma treatment from the perspective of metabolism, especially in the mitochondria. Little is known about the relationship between glioblastoma and mitochondria. There are some reports on glucose starvation and glioblastoma. One report indicated that under glucose starvation, SHC adaptor protein 3 and glucose transporters of the solute carrier 2A superfamily were elevated for adaptation [39]. Other reports suggested that glioblastoma cell metabolic response drives the tricarboxylic acid cycle to deal with glucose starvation [40]. Nonetheless, there are no reports on the relationship between glucose starvation and COX expression. This study elucidated that glucose starvation elevated COX expression and mitochondrial OXPHOS.

According to previous reports, the cancer environment is hypoglycemic [41]. Amino acids and glucose are depleted in tumor cores compared with normal tissues, particularly in poorly vascularized areas [41]. Most nutrient-deprived tissues might have a necrotic core, and hypoxic and nutrient-deficient tissues might also develop malignancy and therapeutic resistance [41, 42]. Tanaka et al. reported that the glioblastoma center is a hypoglycemic environment using gas chromatography-mass spectrometry [41]. In addition, it has been shown that, in a low-nutrient environment, cancer cells become OXPHOS-dominant [17]. Therefore, glucose starvation reflects the tumor environment in vivo, and targeting mitochondria may be an important strategy for treating glioblastoma. Although glioblastoma usually shows increased uptake of fluorine-18-fluorodeoxyglucose-PET [43], considering these reports, intratumor environments may have low glucose concentrations under resting conditions.

Furthermore, we demonstrated that glucose starvation made tumor cells dependent on mitochondria. This implied that mitochondrial inhibition might be effective in achieving tumor cell death. A few studies have reported the use of antibiotics to inhibit mitochondria in cancer cells [18, 26]. However, there are no reports on treatment using medicines that induce mitochondrial dysfunction under glucose starvation. We believe that glucose starvation is important to target mitochondria because starvation makes cells OXPHOS-dominant and this led us to hypothesize that glucose starvation would enhance the effect of antibiotic treatment, which was tested in this study. We found that under glucose-starved conditions, CAP is extremely effective compared with its effect under normal or high glucose conditions.

In addition, we investigated how CAP induces cell death. There are many types of cell death including apoptosis, necroptosis, and cuproptosis [44]. Here, we excluded these types of cell death and found the occurrence of ferroptosis. Among the pathways underlying ferroptosis, the KEAP1–NRF2–HO-1 pathway was altered by CAP in both cell lines. The use of CAP downregulated KEAP1 expression. NRF2 was also elevated because KEAP1 inhibits NRF2 expression [24, 33, 34, 45]. In addition, HO-1 level was elevated via upregulated NRF2 expression [21]. The role of HO-1 is controversial. It acts as an antioxidant and a ferroptosis inducer. Chiang et al. reported that HO-1 exerts a cytoprotective effect by scavenging ROS during moderate activation; in contrast, excessive HO-1 activation increases labile Fe^2+^, leading to ROS overload and cancer cell death [23]. Some authors have also reported that HO-1 promotes ferroptosis after cephem antibiotic injection for nasopharyngeal carcinoma [26]. Here, the results suggested that HO-1 also promotes ferroptosis at a concentration of 100 μM CAP.

Our findings showed that CAP is effective under glucose starvation, and it also downregulates KEAP1 and upregulates NRF2 and HO-1 expression. In addition, we investigated why KEAP1 was downregulated after CAP injection. p62 reportedly inhibits KEAP1, and we hypothesized that p62 was elevated after CAP treatment [24, 33, 34, 45]. Our results showed that p62 was not elevated after CAP injection. However, p-p62 expression, including ser 351 and ser 403, was elevated. Ichimura et al. reported that p-p62 regulates KEAP1 expression. Moreover, p62 is converted to p-p62 and then moves to the autophagosome [35]. This pathway was observed in the U87 and LN229 cell lines. Therefore, we suggest that KEAP1 was downregulated because CAP upregulated p-p62 expression. Figure 6E shows the scheme of the novel mechanism proposed. There are reports of interaction between p-p62 and factors including AMPK and ULK1 [46]. In our study, there was no apparent change in AMPK with CAP. Instead, p-ULK1 (Ser757) was observed to decrease drastically. Kim et al. reported that p-ULK1 (ser 757) inhibits interaction between ULK1 and AMPK and that it inactivates ULK1 [47]. Therefore, we believe ULK1 is activated by CAP. Regarding the relationship between ULK1 and p-p62, Ro et al. reported that Ser 403 of p62 is phosphorylated by ULK1 [48]. Based on this, our findings suggest that p-p62 was increased by activation of ULK1 with CAP. However, the mechanism behind the reason for p62 phosphorylation is complicated and remains unclear. Therefore, further detailed studies are required in future. Mitophagy is reported to be regulated by PINK1/Parkin and LC-3 is changed downstream of this pathway [49–51]. In our results, although no apparent change in PINK1 expression was observed, LC-3 expression was decreased, and we consider the possibility of a decrease in autophagy. Recently, other pathways including AMPK/ULK1 axis that regulate mitophagy were found [49]. We believe there is another mechanism of LC-3 decrease, which needs further detailed studies in the future.

Furthermore, we found that CAP induced ferroptosis using LipiRADICAL Green. In previous studies, fluorescence-activated cell sorting or qPCR was used to detect ferroptosis. According to He et al., antibiotics induced cell death via ferroptosis by altering only HO-1 mRNA level [26]. Herein, we provide a more solid line of evidence for CAP-induced ferroptosis as described below. Ferroptosis is a type of regulated necrosis mainly caused by iron-mediated lipid peroxidation [37]. Therefore, we used LipiRADICAL Green as the lipid radical marker and two inhibitors of ferroptosis to verify this pathway. TFRC expression was elevated after administration of CAP, which implied CAP changed the intracellular iron level. FerroOrange detected intracellular Fe^2+^, which is reported as the causative substance. Moreover, FerroOrange revealed that this hypothesis is true.

Here, we showed that glucose starvation caused cell death after CAP injection, and elucidated its mechanism. Our findings could promote the development of more efficient treatments, for example, combined treatment, including chemoradiotherapy, glucose control, and mitochondrial dysfunction. Regarding the adverse effects of CAP, Dunkle et al. reported that CAP was effective and safe for premature babies with central nervous system infection caused by fungus resistant to penicillin [52]. The clinically used effective blood concentration is 46–154 μM. Here, 10–100 μM CAP was used, which is within the clinically applicable level. Moreover, compared to vancomycin and meropenem used in meningitis treatment, CAP can easily cross the blood–brain barrier [53].

Postoperative infection in patients with glioblastoma reportedly results in a better prognosis [4], but the reason is unclear. We postulate two mechanisms: local immunity and/or the use of antibiotics inhibit tumor growth. We stress that our findings suggested that antibiotics can be one of the effective treatments for glioblastoma. Furthermore, it has been reported that high glucose concentrations promoted glioblastoma cell growth [1]. Tieu et al. reported that glycemia is an independent predictor of survival in patients with glioblastoma treated with RT and TMZ [54]. These reports showed the importance of blood sugar control.

One limitation of this study is that the data were collected in vitro, rather than in vivo. To assess the effectiveness of our treatment in vivo, we used patient-derived stem-like cells that form spheres mimicking tumors. The result obtained with cell lines, that is, CAP is effective under glucose starvation, was also obtained in patient-derived CSC. However, further in vivo investigation using mice is needed. Another limitation of the study was that under the glucose-starved condition, glucose concentration was extremely low. To achieve the effects, 100 mg/l glucose, which was sufficient for cell growth, was selected. To consider for clinical trials, suitable in vivo studies are further required.

In conclusion, we observed an important phenomenon for glioblastoma treatment. In particular, if the current poor prognosis of glioblastoma is taken into consideration, we believe that our findings offer a promising approach for the treatment of glioblastoma.

## Materials and methods

Reagents and antibodies used are shown in Supplementary Table 1.

### Cell culture

U87, LN229, U373, and T98G were obtained from ATCC (Manassas, VA, USA) (all were certified by BEX (Janan)). Cells were cultured in Dulbecco’s modified Eagle’s medium (DMEM, Nacalai Tesque, Japan) containing 10% fetal bovine serum (Sigma-Aldrich, USA) and 1% penicillin–streptomycin (Nacalai Tesque). Cells were cultured in a humidified incubator with 95% air and 5% CO_2_ at 37 °C. Two original patient-derived glioblastoma cell lines, KNS1435 and KNS1451, obtained from Kyushu University Brain Tumor Bank, were suspended in DMEM/ham F12 (Nakarai Tesque), containing human FGF (R&D, USA), EGF (R&D), leukemia inhibitory factor (LIF, Millipore, USA), B27 (Gibco, USA), and penicillin/streptomycin, and plated on a non-attach dish [55]. Genetic analysis revealed that KNS1451 harbors mutations, such as PTEN, TP53, NF1, and TERT promoter C250T and that KNS1435 harbors mutations, such as PTEN, TP53, TERT promoter C228T, PIK3R1, and NOTCH1.

### Quantitative real-time PCR

The total RNA was extracted from cell lines using the RNeasy Mini Kit (QIAGEN, Germany). According to the manufacturer’s instructions, the RNA samples were reverse-transcribed using the PrimeScript RT Reagent Kit (TAKARA, Japan). mRNA expression was detected using qPCR with a thermal cycler (Step One plus; Applied Biosystems). Ribosomal 18S rRNA was evaluated as an internal control. Primer sequences are shown in Supplementary Table 2.

### Immunoblotting analysis

The cells were homogenized in lysis buffer (20 mM Tris-HCL, 2 mM EDTA, 150 mM NaCl, and 1% NP40; pH 7.5), which contained protease (FUJIFILM WAKO, 161-26021, Japan) and phosphatase inhibitors (Sigma-Aldrich, 4906837001). After sonication, the cell lysates were centrifuged at 15,000 × rpm for 5 min. The supernatants were collected as samples. Equal amounts of protein (5 μg) were separated using SDS-PAGE and transferred onto Immobilon-P transfer membranes (EMD Millipore Corporation, Germany). The membranes were blocked using Blocking One (Nacalai Tesque) and then probed overnight with primary antibodies. The membranes were incubated with secondary antibodies. Proteins were detected by enhanced chemiluminescence (GE Healthcare, UK). Chemiluminescence was recorded and quantified using a chilled-charge-coupled device camera (LAS1000plus).

### Immunostaining

Cells seeded in glass bottom dishes (when observed after 24 or 48 h, 2 × 10^4^ cells were seeded; after 72 h, 1 × 10^4^ cells were seeded) were stained with LipiRADICAL Green (1 μM) for 10 min, FerroOrange (1 μM) for 30 min, DQ-BSA (50 μg/ml) for 60 min, or Mito Tracker Red (100 nM) for 20 min at 37 °C. The medium was replaced with HBSS (FUJIFILM WAKO, 084-08965) before staining. The intensities of the signals were measured and calculated (calculated average luminance (total luminance/cell number) in each image (*N* = 10) (BZ-X800; KEYENCE).

### Cell viability

Cells (1 × 10^4^ in 12-well dish, 1 × 10^5^ in 6-well dish) were seeded in triplicate and cultured in DMEM (containing glucose, CAP, and inhibitors at each concentration). The cells were trypsinized and counted daily for up to 72 h using a Coulter counter (Beckman Coulter, USA) and TC 20 automated cell counter (BIO-RAD, #1450101J1, USA) with trypan blue. For the assessment of the proliferation of stem-like cells, the cells (2 × 10^4^ in 6-well dish) were seeded in triplicate or more and cultured in DMEM/ham F12 (containing glucose and CAP at each concentration) for 7 days (9 days in KNS1435). After centrifugation at 3000 *×* *g* for 3 min, the cell pellets were trypsinized, added to the medium, and then counted using TC 20 cell automated cell counter with trypan blue.

### Seahorse XF24 flux analyzer

Mitochondrial OXPHOS and glycolytic activity can be measured using the OCR and extracellular acidification rate (ECAR) methods with an XFe24 Analyzer (Seahorse Biosciences, USA). Seahorse XF24 microplates were seeded with 1 × 10^5^ cells/well (before seeding, cells were incubated for 2 or 6 days under each glucose condition) and incubated at 37 °C for ~1 day. Basal OCR and ECAR were measured using the Seahorse XF24 Flux analyzer. Additional measurements were performed after injecting compounds affecting bioenergetics: oligomycin, 0.75 mM; carbonyl cyanide 4-trifluoromethoxyphenylhydrazone, 500 nM; 2-deoxyglucose, 50 mM; glucose, 25 mM; and rotenone and antimycin, 1 μM (all from Sigma-Aldrich). After analysis, the cells were trypsinized, counted, and the results were normalized to the number of cells.

### Statistical analysis

Statistical analyses are described in figure legends. Data are presented as mean ± SD. Significant differences between groups were examined using one-way ANOVA or Student’s *t* test with GraphPad Prism 9 (GraphPad Prism Software Inc). All experiments were repeated at least three times.

## Supplementary information

supplemental material

## Data Availability

All data generated or analyzed during this study are included.
