# Correction: Mitochondrial dysfunction and impaired growth of glioblastoma cell lines caused by antimicrobial agents inducing ferroptosis under glucose starvation

**DOI:** 10.1038/s41389-022-00443-1

**Published:** 2022-10-19

**Authors:** Kenji Miki, Mikako Yagi, Koji Yoshimoto, Dongchon Kang, Takeshi Uchiumi

**Affiliations:** 1 Department of Clinical Chemistry and Laboratory Medicine, Graduate School of Medical Sciences, Kyushu University, Higashi-ku, Fukuoka, 812-8582 Japan; 2 Department of Neurosurgery, Graduate School of Medical Sciences, Kyushu University, Higashi-ku, Fukuoka, 812-8582 Japan; 3 Department of Health Sciences, Graduate School of Medical Sciences, Kyushu University, Higashi-ku, Fukuoka, 812-8582 Japan

**Keywords:** CNS cancer, Apoptosis

Correction to: *Oncogenesis* 10.1038/s41389-022-00437-z, published online 04 October 2022

In Figure 5G, was written by mistake CAP+ZnPPIX description left of the picture located bottom.

however, this was a mistake, and correct version is CAP+DFO.

The original article has been corrected.

