# Supplementary material for: Mitochondrial dysfunction and impaired growth of glioblastoma cell lines caused by antimicrobial agents inducing ferroptosis under glucose starvation
Source: Oncogenesis. 2022 Oct 4;11(1):59. doi: 10.1038/s41389-022-00437-z (PMC9532440; doi:10.1038/s41389-022-00437-z)
Supplement: Supplementary file 1 — supplemental material [file 41389_2022_437_MOESM1_ESM.pdf]

Supplementary Figure 1

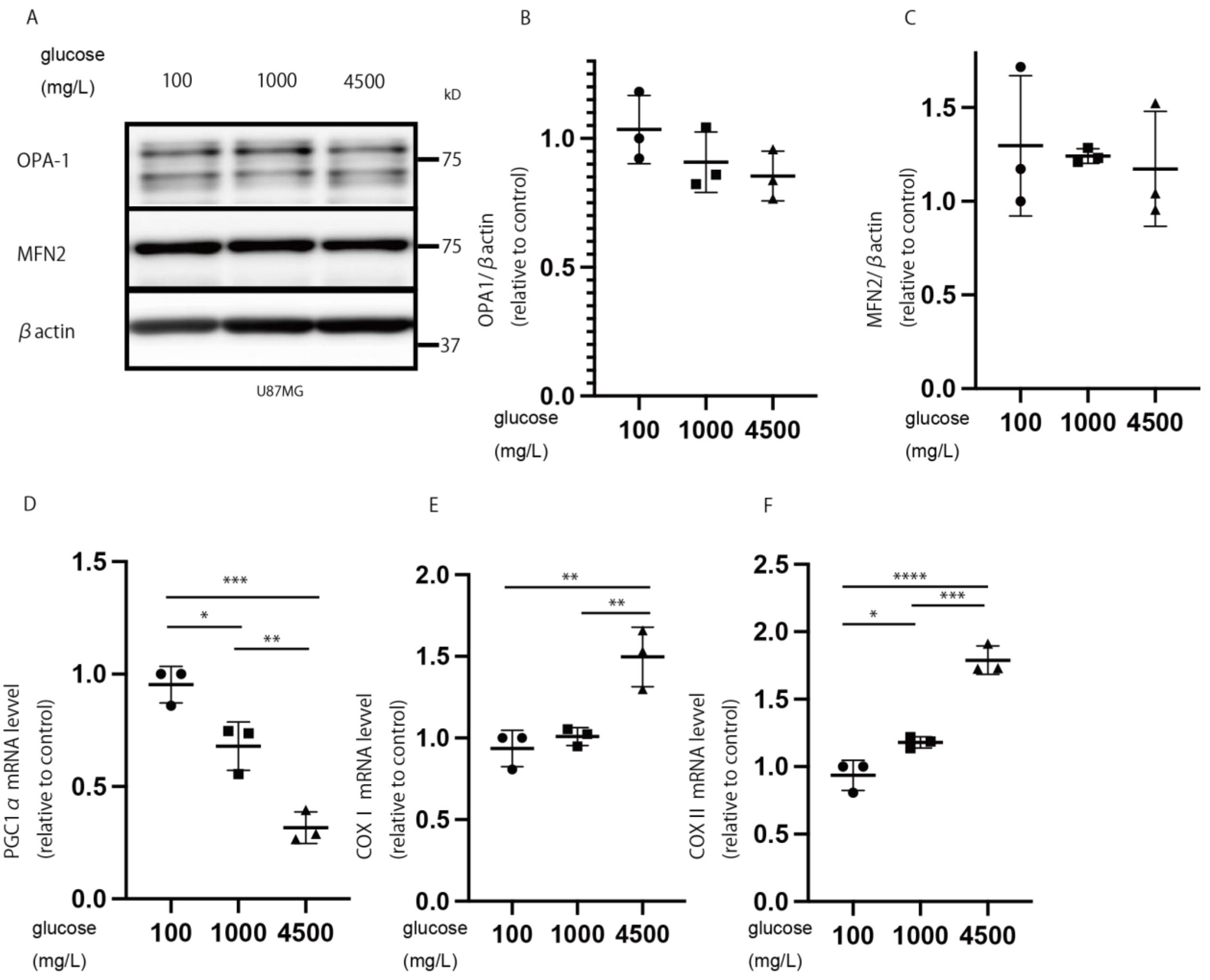

Supplementary Figure 2

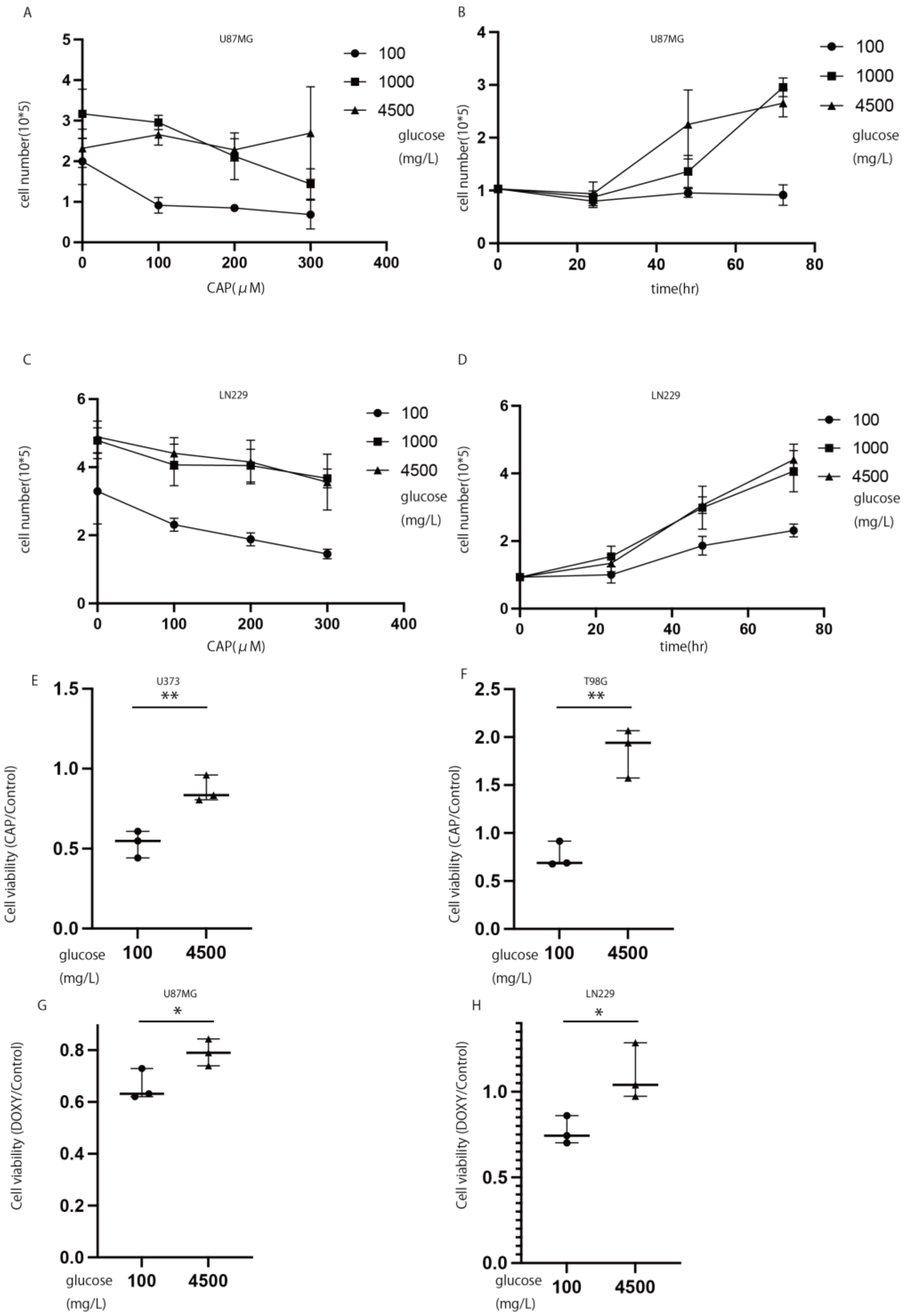

Supplementary Figure 3

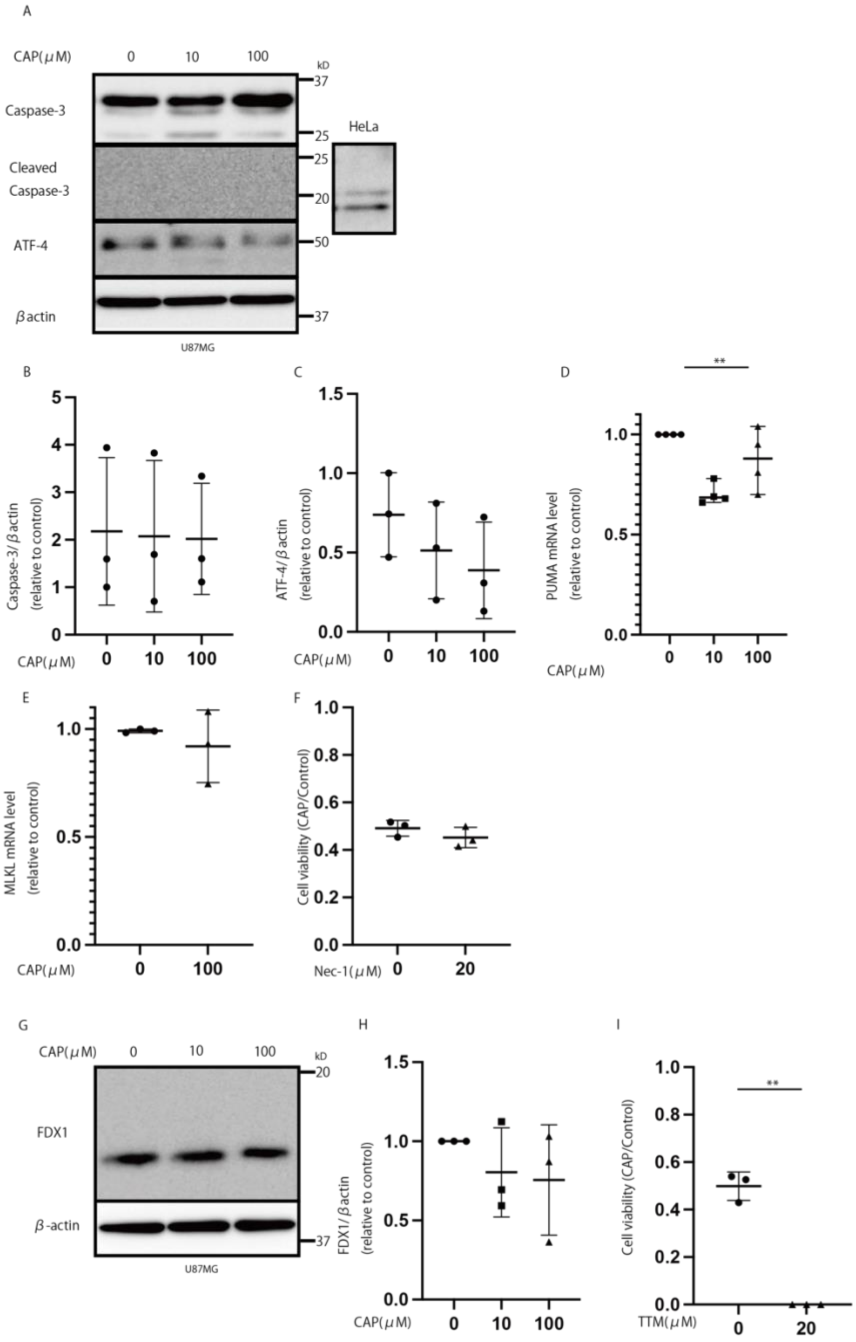

Supplementary Figure 4

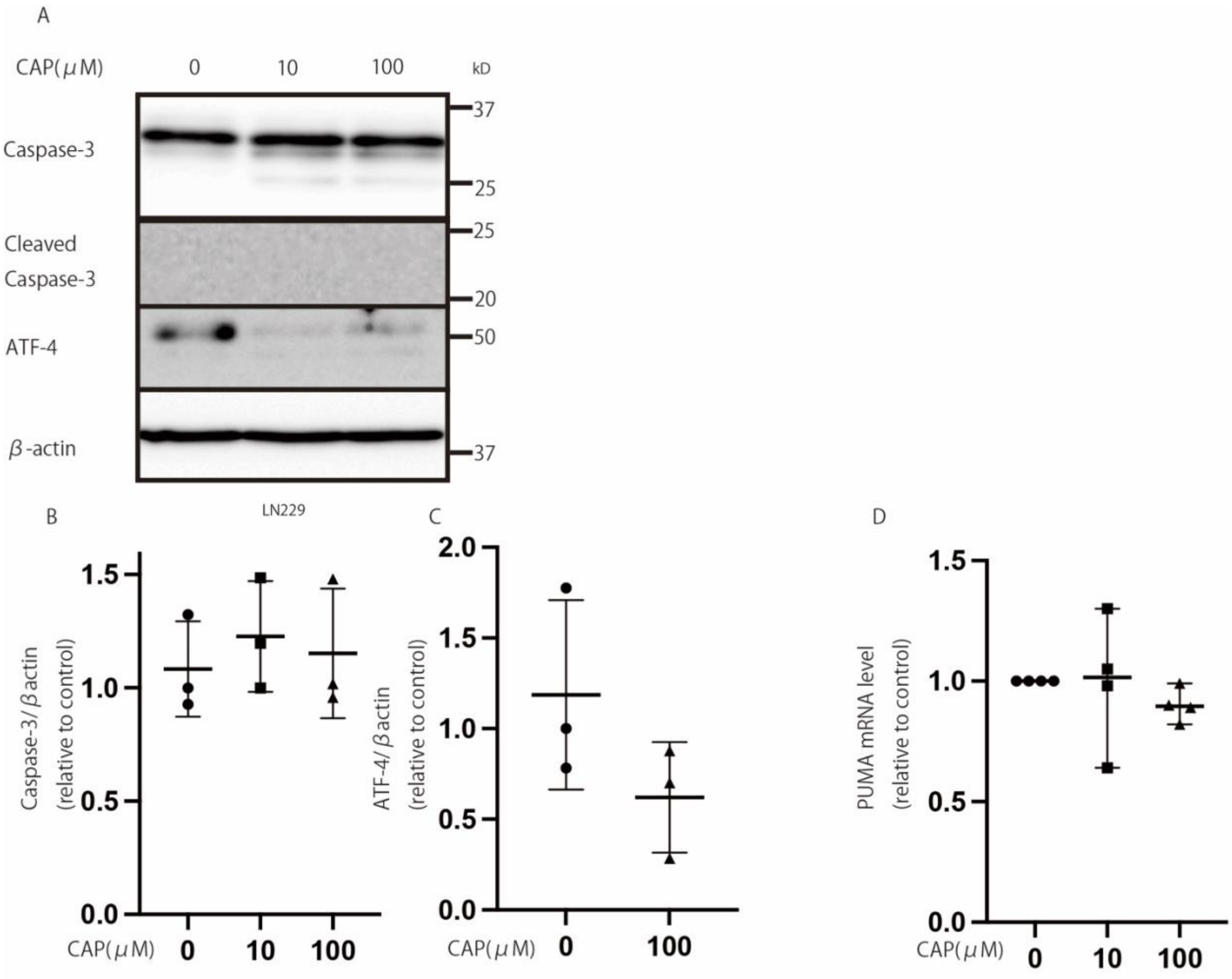

Supplementary Figure 5

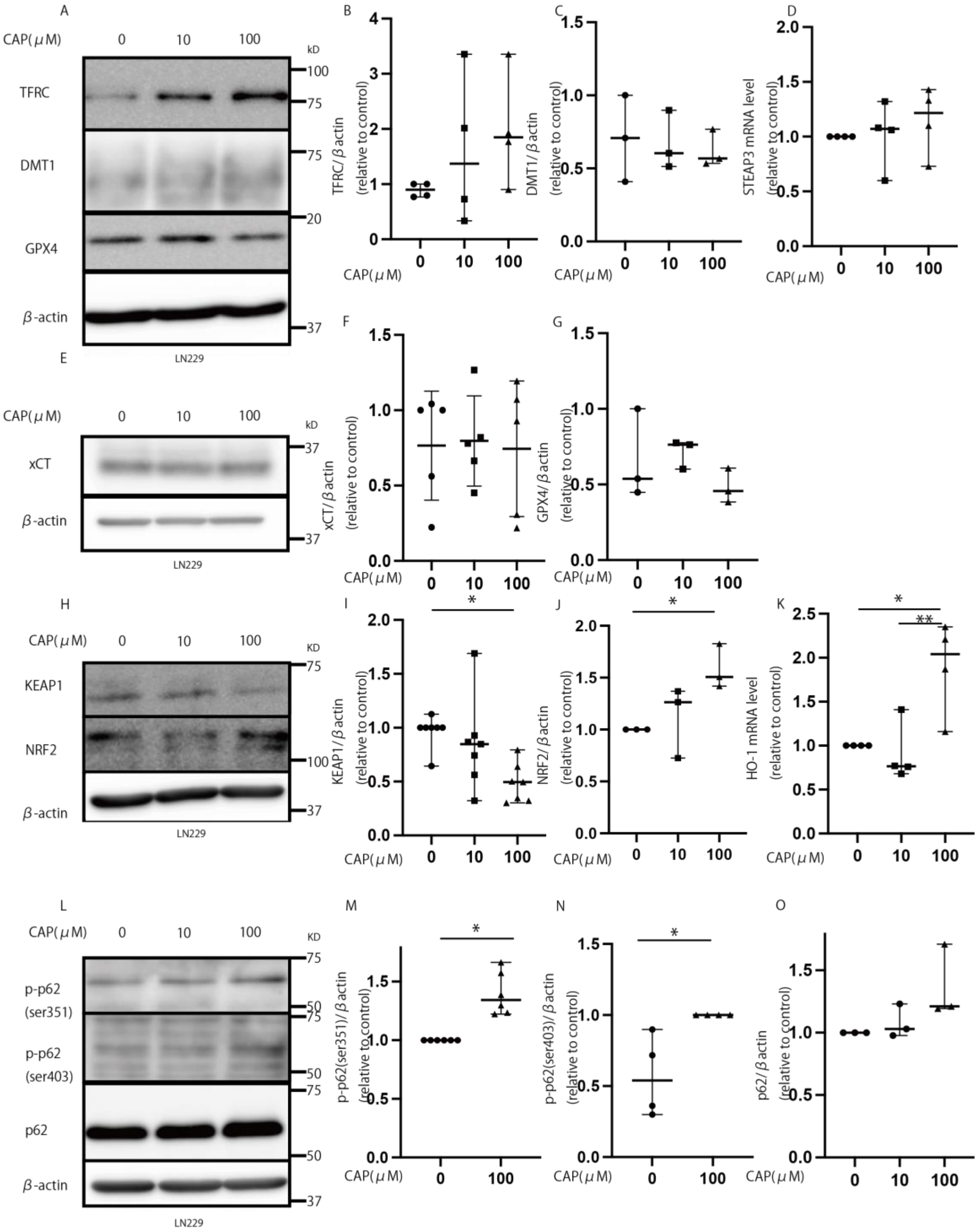

Supplementary Figure 6

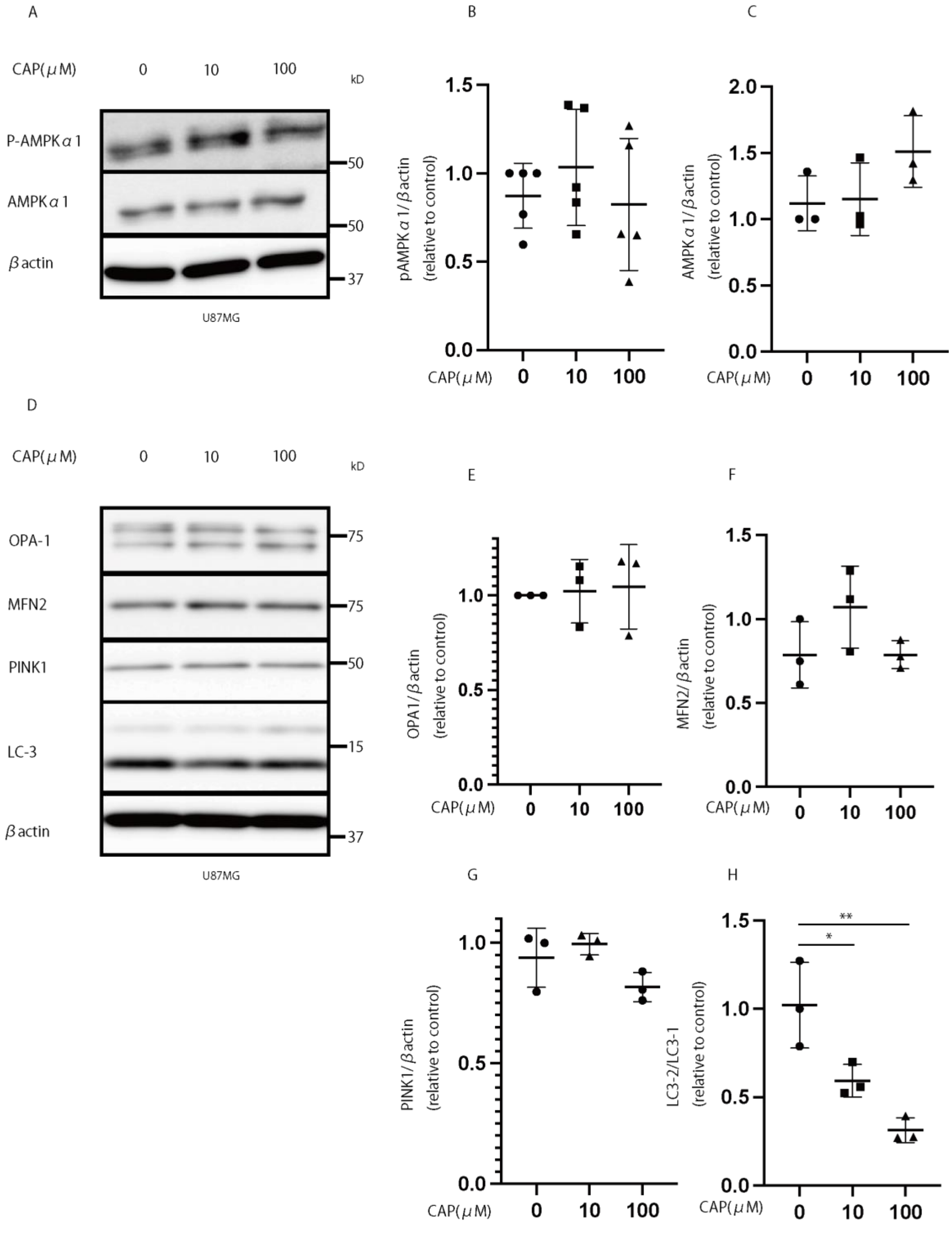

Supplementary Figure 7

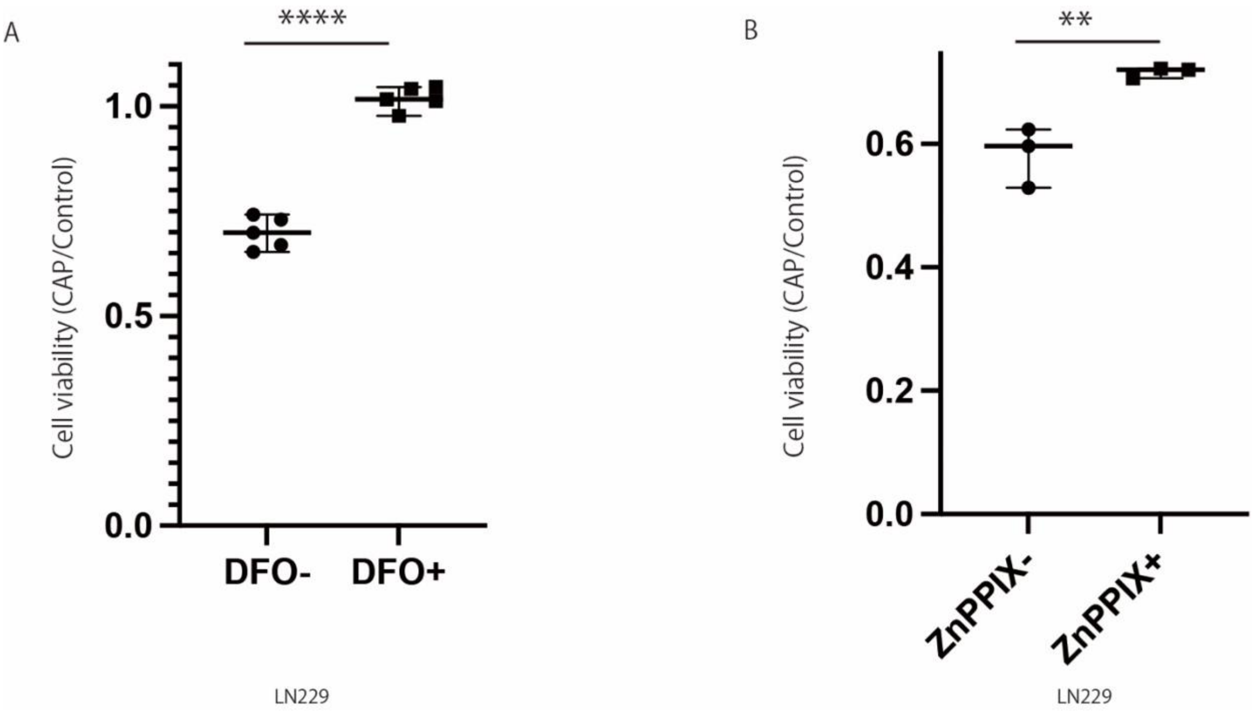

Supplementary Figure S1. Effects of glucose on morphology and OXPHOS mRNA.

(A) Western blot of OPA1 and MFN2 in U87 under glucose starvation after 7-day culture. β-Actin was used as an internal control. (B, C) Quantification of OPA1 and MFN2 in U87. (D-F) mRNA of PGC1α, mt-coded COX I and COX II in U87 under glucose starvation after 7-day culture. Quantification of (D) *PGC1α*, (E) *COX I*, and (F) *COX II* mRNA expression (N = 3). Values are presented as mean ± SD. Ordinary one-way ANOVA with Tukey's multiple comparisons test was performed on control vs. CAP 10 μM and control vs. CAP 100 μM. *$p < 0.05$, **$p < 0.01$, ***$p < 0.001$, ****$p < 0.0001$.

Supplementary Figure S2. Effects of antimicrobial agents under glucose-starved conditions.

(A) Relationship between CAP and glucose concentration in U87. Growth curve of CAP dose-dependency and each glucose condition after 3-day culture (cells were seeded in a 12-well dish and counted using a Coulter counter) (N = 3 or 4). (B) Time-course of growth curve with CAP 100 μM with each glucose concentration (N = 3 or 4). (C) Relationship between CAP and glucose concentration in LN229. Survival curve of CAP dose-dependency and each glucose condition after 3-day culture (cells were seeded in a 6-well dish and counted using trypan blue) (N = 3). (D) Time-

course of survival curve with CAP 100 μM with each glucose concentration (N = 3). (E) Cell viability under CAP 100 μM treatment with each glucose concentration in U373 (cells were seeded in a 6-well dish and counted using a Coulter counter) (N = 3). (F) Cell viability under CAP 100 μM treatment with each glucose concentration in T98G (cells were seeded in a 6-well dish and counted using a Coulter counter) (N = 3). (G) Relationship between DOXY and glucose concentration in U87. Cell viability under DOXY 100 μM treatment with each glucose concentration (cells were seeded in a 6-well dish and counted using a Coulter counter) (N = 3). (H) Cell viability under DOXY 100 μM treatment with each glucose concentration in LN229 (cells were seeded in a 6-well dish and counted using a Coulter counter) (N = 3). Values are presented as mean ± SD. Student's *t*-test was performed. *p < 0.05. **p < 0.01.

Supplementary Figure S3. Apoptotic pathways are not activated in U87. CAP was added to U87 culture for 3 days under glucose starvation (glucose 100 mg/l). (A) Western blot of caspase-3, cleaved caspase-3, and ATF-4. Positive control of cleaved caspase-3 was HeLa cells. (B) Quantification of caspase-3 (N = 3) and (C) ATF-4 (N = 3). (D) Quantification of *PUMA* mRNA expression (N = 4). (E) Quantification of *MLKL* mRNA expression (N = 3). (F) Cell viability assay

revealed that CAP and Nec-1 did not reduce cell death after 3-day culture. Nec-1 was injected at a concentration of 20 μM initially (N=3). (G) Western blot of FDX1 and (H) quantification (N=3). (I) Cell viability assay revealed that CAP and TTM did not reduce cell death after 3-day culture. TTM was injected at a concentration of 20 μM initially (N=3). Values are presented as mean ± SD. Student's *t*-test or ordinary one-way ANOVA with Tukey's multiple comparisons test was performed on control vs. CAP 10 μM and control vs. CAP 100 μM. **$p < 0.01$

Supplementary Figure S4. Apoptotic pathways are not activated in LN229. CAP did not cause apoptosis under glucose starvation (glucose 100 mg/l). (A) Western blot of caspase-3, cleaved caspase-3, and ATF-4. Quantification of (B) caspase-3 and (C) ATF-4 (N = 3). (D) Quantification of *PUMA* mRNA expression (N = 4). Values are presented as mean ±SD. Student's *t*-test (C) or ordinary one-way ANOVA test (B,D) was performed on control vs. CAP 10 μM and control vs. CAP 100 μM.

Supplementary Figure S5. KEAP 1-NRF 2-HO-1 pathways are activated in LN229. Ferroptosis was changed with CAP treatment after 3-day culture. (A) Western blot revealed that

TFRC (N = 4) expression tended to increase, but DMT1 (N = 3) expression did not change after CAP treatment and (B, C) quantification. (D) Quantification of *STEAP3* mRNA expression (N = 4). (E) Western blotting of xCT (N=5) and (F) quantification. (G)Quantification of GPX4. (H) Western blotting revealed that KEAP1 (N = 7) and NRF2 (N = 3) expression was changed and (I, J) quantification results. (K) Quantification of *HO-1* mRNA expression (N = 4). (L) Western blot of p-p62 ser 351(N = 5), 403 (N = 4), and p62 (N = 3) after CAP treatment and (M-O) quantification results. Values are presented as mean ±SD. Student's *t*-test (M,N) or ordinary one-way ANOVA with Tukey's multiple comparisons test (B-D, F-G, I-K, O) was performed on control vs. CAP 10 μM and control vs. CAP 100 μM. *p < 0.05, **p < 0.01.

Supplementary Figure S6. Effects of CAP on mitochondrial morphology and mitophagy under glucose starvation condition.

CAP did not change mitochondrial morphology but altered mitophagy under glucose-starved conditions (glucose 100 mg/l). (A) Western blot of pAMPKα1 and AMPKα1. Quantification of (B) pAMPKα1 (N = 5) and (C) AMPKα1 (N = 3). (D) Western blot of OPA1, MFN2, PINK1, and LC-3. Quantification of (E) OPA-1, (F) MFN2, (G) PINK1, and (H) LC3 (N = 3). Values are

presented as mean ±SD. Ordinary one-way ANOVA test was performed on control vs. CAP 10 μM and control vs. CAP 100 μM. *$p<0.05$, **$p<0.01$.

Supplementary Figure S7. Inhibition of ferroptosis by DFO and ZnPPIX in LN229. (A, B) Cell viability assay revealed that CAP and DFO or ZnPPIX reduced cell death after 3-day culture. DFO was injected at 50 μM initially, and then 25 μM was injected at 48 h, and ZnPPIX was injected at 2.5 μM initially. Values are presented as mean ±SD. Student's *t*-test was performed (N = 3). **$p < 0.01$, ****$p < 0.0001$.

Supplementary Table 1

antibodies	catalog number	Source
anti-NDUFA9 mouse mAb	#ab14713	Abcam
anti-SDHA mouse mAb	#ab14715	Abcam
anti-Complex3 mouse mAb	#ab110252	Abcam
anti-COX1(MTCO1) mouse mAb	#ab14705	Abcam
anti-COX2(MTCO2) mouse mAb	#ab110258	Abcam
anti-ATP5A mouse mAb	#ab14748	Abcam
anti-PINK1 mouse mAb	#ab75487	Abcam
anti-FDX1 rabbit mAb	#ab108257	Abcam
anti-cleaved caspase-3 rabbit mAb	#9664	CST
anti-caspase-3 rabbit mAb	#9662	CST
anti-ATF4 rabbit mAb	#11815	CST
anti-DMT1/SLC11A2 rabbit mAb	#15083	CST
anti-GPX4 rabbit pAb	#52455	CST
anti-KEAP1 rabbit mAb	#8047	CST
anti-NRF2 rabbi mAb	#12721	CST
anti-xCT/SLC7A11 rabbit mAb	#12691	CST
anti-phospho-AMPKα1 rabbit mAb	#2535	CST
anti-AMPKα1 rabbit mAb	#2603	CST
anti-MFN2 rabbit mAb	#9482	CST
anti-LC3A/B rabbit mAb	#12741	CST
anti-ULK1 rabbit mAb	#8054	CST
anti-phospho-ULK1(S757) rabbit pAb	#6888	CST
anti-mouse IgG HRP-linked	#7076	CST
anti-rabbit IgG HRP-linked	#7074	CST
anti-rat IgG HRP-linked	#7077	CST
anti-OPA1 mouse mAb	#612606	BD Biosciences
anti-β-actin mouse mAb	#A5441	Sigma-Aldrich

anti-TFRC mouse mAb	#13-6800	Thermo Fisher Scietific
anti-p62(SQSTM1) rabbit pAb	#PM045	MBL
anti-phospho-p62(SQSTM1)(Ser351) mouse mAb	#PM074	MBL
anti-phospho-p62(SQSTM1)(Ser403) rat mAb	#D343-3	MBL
chemicals, enzymes and other reagents		
CAP	034-10572	WAKO
DOXY	D9891-5G	Sigma-Aldrich
Mito Tracker Red	M7512	Invitrogen
LipiRADICAL Green	FDV0042	FNA
Ferro Orrange	F374	DOJINDO
DQ-BSA	D12050	Thermo Fisher Scientific
Necrostatin-1	HY15760	MedChemExpress
Ammonium Tetrathiomolybdate	013-26932	WAKO
Deferoxamine	205-314-3	Sigma-Aldrich
protoporphiryn IX zinc	14483	CAY

Abcam (Cambridge, UK), Cell Signaling Technology (CST, Danvers, MA, USA), BD Biosciences (NJ, USA), MBL (Tokyo, Japan), FUJIFILM WAKO (WAKO, Osaka, Japan), Invitrogen (Carlsbad, CA, USA), FNA (Tokyo, Japan), DOJINDO (Kumamoto, Japan), Thermo Fisher Scientific (Waltham, MA, USA), MedChemExpress (NJ, USA), Sigma-Aldrich (St. Louis, MO, USA), CAY (MI, USA).

Supplementary Table 2

target	forward(5'→3')	reverse(3'→5')
PUMA	GAGATGGAGCCCAATTAGGTG	TCCAGTATGCTACATGGTGCAG
STEAP3	AAAACCACACTGGCTCCAAC	GTCAGTAGGGAGCAGCAAGG
HO-1	GGCCTCCCTGTACCACATCT	AGACAGGTCACCCAGGTAGC
18S	AAACGGCTACCACATCCAAG	CCTCCAATGGATCCTCGTTA
PGC1α	AGCTGCTGAAGAGGCAAGAG	TTCCCCTAAACCAAGCACAC
COX1	GGCCTGACTGGCATTGTATT	TGGCGTAGGTTTGGTCTAGG
COX2	TTCATGATCACGCCCTCATA	TAAAGGATGCGTAGGGATGG
MLKL	CTCTTTCCCCACCATTTGAA	TCATTCTCCAGCATGCTCAC
